# The impact of different inclusion decisions on the comprehensiveness and complexity of overviews of reviews of healthcare interventions

**DOI:** 10.1186/s13643-018-0914-3

**Published:** 2019-01-11

**Authors:** Michelle Pollock, Ricardo M. Fernandes, Amanda S. Newton, Shannon D. Scott, Lisa Hartling

**Affiliations:** 1 Department of Pediatrics, Alberta Research Centre for Health Evidence, University of Alberta, Edmonton, Canada; 2 Clinical Pharmacology Unit, Instituto de Medicina Molecular, University of Lisbon, Lisbon, Portugal; 3 Department of Pediatrics, Santa Maria Hospital, Lisbon, Portugal; 4 Department of Pediatrics, University of Alberta, Edmonton, Canada; 5 Faculty of Nursing, University of Alberta, Edmonton, Canada; 6 4-472 Edmonton Clinic Health Academy, 11405 87 Avenue NW, Edmonton, AB T6G-1C9 Canada

**Keywords:** Overview of reviews, Systematic review, Knowledge synthesis, Case series

## Abstract

**Background:**

Overviews of reviews (overviews) compile information from multiple systematic reviews (SRs) to provide a single synthesis of relevant evidence for decision-making. Overviews may identify multiple SRs that examine the same intervention for the same condition and include some, but not all, of the same primary studies. There is currently limited guidance on whether and how to include these overlapping SRs in overviews. Our objectives were to assess how different inclusion decisions in overviews of healthcare interventions affect their comprehensiveness and results, and document challenges encountered when making different inclusion decisions in overviews.

**Methods:**

We used five inclusion decisions to conduct overviews across seven topic areas, resulting in 35 overviews. The inclusion decisions were (1) include all Cochrane and non-Cochrane SRs, (2) include only Cochrane SRs, or consider all Cochrane and non-Cochrane SRs but include only non-overlapping SRs, and in the case of overlapping SRs, select (3) the Cochrane SR, (4) the most recent SR (by publication or search date), or (5) the highest quality SR (assessed using AMSTAR). For each topic area and inclusion scenario, we documented the amount of outcome data lost and changed and the challenges involved.

**Results:**

When conducting overviews, including only Cochrane SRs, instead of all SRs, often led to loss/change of outcome data (median 31% of outcomes lost/changed; range 0–100%). Considering all Cochrane and non-Cochrane SRs but including only non-overlapping SRs and selecting the Cochrane SR for groups of overlapping SRs (instead of the most recent or highest quality SRs) allowed the most outcome data to be recaptured (median 42% of lost/changed outcome recaptured; range 28–86%). Across all inclusion scenarios, challenges were encountered when extracting data from overlapping SRs.

**Conclusions:**

Overlapping SRs present a methodological challenge for overview authors. This study demonstrates that different inclusion decisions affect the comprehensiveness and results of overviews in different ways, depending in part on whether Cochrane SRs examine all intervention comparisons relevant to the overview. Study results were used to develop an evidence-based decision tool that provides practical guidance for overview authors.

**Electronic supplementary material:**

The online version of this article (10.1186/s13643-018-0914-3) contains supplementary material, which is available to authorized users.

## Background

Systematic reviews (SRs) of healthcare interventions aim to assess an intervention’s efficacy or effectiveness by using explicit and reproducible methods to combine the results of all relevant primary studies [1]. By synthesizing all available data, SRs attempt to explore and ultimately resolve discrepancies among primary studies that may have different, and sometimes contradictory, results of an intervention’s effect. However, as the number of published SRs steadily increases [2], it is becoming increasingly common to find multiple SRs that address the same, or very similar, clinical questions [3]. We refer to these as “overlapping SRs,” and they may include many, but not necessarily all, of the same primary studies, due to differences in methods used for inclusion criteria, search strategies, study selection, and data extraction and analysis [3].

Researchers conducting overviews of reviews of healthcare interventions (overviews) often encounter overlapping SRs. Overviews use explicit and systematic methods to compile data from multiple, related SRs to provide a single synthesis of evidence for healthcare decision-making [4]. They are typically broader in scope than any individual SR and often examine the efficacy or effectiveness of multiple interventions for preventing or treating a specific clinical condition [4]. Overview authors can encounter overlapping SRs when they decide to include both SRs published in and outside of the Cochrane Database of Systematic Reviews (“Cochrane SRs” and “non-Cochrane SRs”). This is because Cochrane attempts to avoid duplication of effort by publishing only one SR on healthcare interventions for a specific condition or illness, whereas multiple non-Cochrane SRs can exist to address the same, or very similar, clinical questions. Researchers that choose to include overlapping SRs in overviews will encounter important methodological considerations [5–8]. Overview authors should properly assess the amount of overlap in the primary studies contained within the overview’s included SRs. If overlap exists, and the outcome data from some primary studies contribute more than once to the analyses, bias can be introduced into the overview as disproportionate weight has been given to some of the data [5]. Researchers may also find it difficult to appropriately extract and analyze outcome data from overlapping SRs if their conduct, quality, and/or reporting differs between SRs [6]. Further, if overlapping SRs included in the overview have discordant results and/or conclusions, researchers need to decide how they will synthesize and discuss these differences [6]. Despite these methodological considerations, only half of the overviews that contain overlapping SRs currently acknowledge and discuss the overlap [5].

To date, researchers have used several approaches to manage overlapping SRs in overviews [6, 7]. Some researchers have included all relevant Cochrane and non-Cochrane SRs and avoided overlap by extracting outcome data for each primary study only once (regardless of how many SRs contained that study’s data) [9, 10]. Others have avoided overlap by restricting the overview to synthesizing only Cochrane SRs [4, 8, 11], while others have included Cochrane and non-Cochrane SRs and avoided overlap by using specific criteria to prioritize SR inclusion when confronted with multiple, overlapping SRs (e.g., only include the Cochrane, most recent, or highest quality SR) [8, 9]. Currently, there is no empirical evidence on the impact of these different inclusion decisions, and no guidance for how to choose one method of inclusion over another [6, 7].

The purpose of this study was to provide empirical evidence examining the inclusion of overlapping SRs in overviews of reviews of healthcare interventions. Specifically, we assessed how different decisions surrounding the inclusion and exclusion of overlapping SRs in overviews affect the comprehensiveness and results of overviews, and documented challenges encountered when using different inclusion criteria in overviews. Results of this study were then used to develop an evidence-based decision tool to help overview authors make inclusion decisions in overviews. This tool is presented in a companion paper by Pollock et al. [12].

## Methods

### Study procedures

This was a multiple case study [13]. Each “case” was an overview of reviews conducted by the Alberta Research Centre for Health Evidence between 2010 and 2016 that examined a question related to the efficacy or effectiveness of multiple healthcare interventions for preventing or treating a clinical condition related to pediatric health. Seven cases [14–20] were included in the study based on convenience sampling [21]: acute asthma [14], acute otitis media [15], bronchiolitis [16], croup [17], eczema [18], gastroenteritis [19], and procedural sedation [20]. The inclusion criteria (populations, interventions, comparators, outcome measures, and study designs) for each case are provided in Additional file 1. For feasibility, we used clinical judgment to restrict the inclusion criteria of four cases, compared to the inclusion criteria used in the published overviews (see footnotes in Additional file 1). We then conducted each of the seven overview cases using five different inclusion decisions (described in detail below). This resulted in 35 overviews of healthcare interventions. We assessed the impact of the different inclusion decisions on the comprehensiveness and results of the overviews, both within and across overview cases.

### Conducting the overviews

For each overview, all published, English-language Cochrane and non-Cochrane SRs that met the overview’s inclusion criteria were identified from its reference list. All seven overviews searched for Cochrane SRs; four also searched for non-Cochrane SRs. For the three overviews that did not search for non-Cochrane SRs [15–17], we conducted additional literature searches to locate and include non-Cochrane SRs that met the overview’s inclusion criteria. An information specialist conducted the literature searches using the inclusion criteria and search dates from each overview (AM). The search strategies for all overview topics are available in published overviews and upon request. Screening non-Cochrane SRs for inclusion was conducted independently by two reviewers, with discrepancies resolved by consensus or third party adjudication (AC, AM, DO, JS, MM, MO). At the end of the literature identification stage, each of the seven overview cases consisted of a published overview along with all published English-language Cochrane and non-Cochrane SRs that met that overview’s inclusion criteria. Two reviewers independently assessed the methodological quality of each SR in each overview using A MeaSurement Tool to Assess systematic Reviews (AMSTAR) [22], with discrepancies resolved via consensus or third party adjudication (MP, LH, AC, AM, IS, MO, SS). AMSTAR scores (/11) were summarized using means and standard deviations.

The seven overview cases were conducted sequentially, according to five different inclusion scenarios, for a total of 35 overviews. The five inclusion scenarios were chosen because they are commonly cited in the literature as potentially appropriate ways to manage overlapping SRs in overviews [6, 7, 9]. The inclusion scenarios guided the decisions on which SRs and outcome data to include in each overview, as follows:

- Full inclusion scenario: All eligible outcome data were extracted from all eligible Cochrane and non-Cochrane SRs. We ensured accuracy of effect estimates by making sure that each primary study’s outcome data were extracted only once (regardless of how many SRs contained that study’s data). This involved extracting data from the Cochrane SR (if present), followed sequentially by the most recent and/or highest quality SRs that most closely matched the overview’s scope for each intervention comparison.
- Restricted scenario 1: All eligible outcome data were extracted from all Cochrane SRs.
- Restricted scenarios 2 to 4: All eligible outcome data were extracted from all non-overlapping SRs, and in the case of groups of overlapping SRs, we included outcome data from the Cochrane SR (restricted scenario 2), most recent SR (restricted scenario 3), or highest quality SR (restricted scenario 4). For restricted scenario 2, if there was no Cochrane SR within a group of overlapping SRs, no outcome data were extracted. For restricted scenario 3, the most recent SR was defined as the SR with the most recent year of publication (for Cochrane SRs, we used the “year last assessed as up-to-date”). If two SRs were tied for “most recent,” we included the one with the most recent search date. For restricted scenario 4, the highest quality SR was defined as the SR with the highest AMSTAR score (/11). If two SRs were tied for “highest quality,” we noted this in the results files and did not extract data, as there are currently no accepted criteria for objectively choosing between two SRs with the same quality scores. It was possible for different restricted scenarios to end up including the same SRs if, for example, the Cochrane SR was also the most recent and/or the highest quality SR.

Matrices showing which comparisons and SRs were included in the overviews are provided in Additional file 2. Because many SRs examined multiple interventions and comparators, we assessed overlap within SRs for each individual comparison.

Data extraction and analysis for the 35 overviews adhered to standard methods [4]. The following data were extracted for each of the 35 overviews: (1) descriptive characteristics of the SRs (e.g., Cochrane or non-Cochrane, first author, year of publication, populations, and included comparisons), (2) descriptive characteristics of the included primary studies contained within the SRs (e.g., first author, year of publication, study design, and sample size, for studies that matched the relevant overview’s inclusion criteria), and (3) outcome data. We extracted outcome data from all relevant primary studies for all primary, secondary, adverse effects, and supplemental outcome measures, as specified in the corresponding overviews (Additional file 1). When raw outcome data were reported in SRs, numerical data were extracted from SRs and re-analyzed in Review Manager 5.3 (The Cochrane Collaboration, Copenhagen, Denmark), using standard meta-analysis techniques [23]. Outcome data were expressed using the measures of effect used in the corresponding overviews (risk ratios, odds ratios, and/or risk differences for dichotomous outcomes, and mean differences and/or standardized mean differences for continuous outcomes), with 95% confidence intervals. We conducted all analyses using random effects modeling and the Mantel-Haenszel method (dichotomous data) or inverse variance method (continuous). When meta-analyzed data were reported in SRs but raw study data were not provided, or when only narrative data were provided, the data were extracted and reported based on statistical significance or the SR authors’ description as “significant in favor of intervention,” “not significant,” or “significant in favor of comparator.” For additional methodological decisions unique to each case, we adhered to the decision rules contained within the “Methods” section of the published overviews (though for feasibility, we did not conduct any subgroup or sensitivity analyses). Outcome data from the SRs contained within the procedural sedation overview case were not extracted, because data for the comparator groups were often not available.

We classified all outcome data using published criteria as “favorable” (*p* ≤ 0.05 in favor of the intervention, or finding described as “significant”), “neutral” (*p* > 0.05, or finding described as “not different between groups”), or “unfavorable” (*p* ≤ 0.05 in favor of the comparator, or finding described as “favoring non-intervention comparator”) [24, 25]. We classified outcome data as “unknown” when the effect estimate was not estimable (due to no events in either group) or when the “full inclusion scenario” contained discordant outcome data from multiple overlapping SRs. One reviewer (MP) extracted and analyzed the data, and two additional reviewers (RMF, LH) oversaw this process and provided clinical and methodological input as needed. One reviewer (MP) also documented the challenges encountered when conducting the overviews according to the different inclusion scenarios, and discussed these challenges with two additional reviewers (RMF, LH).

### Data analysis

Data analysis consisted of both within-case analyses and cross-case syntheses [13, 26]. For each of the seven overview topics, we used three complimentary methods to visualize and describe the “full inclusion scenario”: (1) we reported characteristics of the included SRs and their primary studies; (2) we generated a citation matrix [5] to show which SRs (columns) included which primary studies (rows), with sample sizes of primary studies reported in relevant cells; and (3) we used the citation matrix to calculate the “corrected covered area” (CCA) [5] to assess the extent of primary study overlap between the SRs included in the overview. The CCA represents “the area [of the citation matrix] that is covered after eliminating the inclusion of all primary studies the first time they are counted” [5]. The formula is, CCA = $\frac{N-r}{rc-r}$, where *N* = total number of included primary studies (number of non-empty cells), *r* = total number of unique primary studies (number of rows), and *c* = total number of SRs (number of columns). The amount of overlap could range from 0 to 100 and was categorized using published criteria as “slight” (0–5), “moderate” (6–10), “high” (11–15), or “very high” (> 15). Detailed instructions for creating citation matrices and calculating the CCA can be found in Pieper et al. [5].

For each of the six overview topics for which outcome data were extracted, we systematically compared “restricted scenarios 1 to 4” to the full inclusion scenario and documented the extent of data loss and change. We calculated the number and percentage of SRs, intervention comparisons, primary studies, and subjects that were lost in each restricted scenario. For the overviews’ outcome data, we compared the result classifications obtained in each of the four restricted scenarios to those obtained in the full scenario. Each outcome was described as “no change” (the result classification remained the same in both the restricted and full scenarios), “change” (the result classification differed in the restricted compared to the full scenario), or “data lost” (all data for that outcome were lost in the restricted scenario). We then calculated the number and percentage of primary, secondary, adverse effect, and supplemental outcomes that were lost and changed in each restricted scenario. These data were organized into a case-ordered descriptive matrix to permit within-case and cross-case analyses [13, 26].

As is standard with a multiple case study, we aimed to demonstrate replication logic across cases [13]. We summarized the effects of the five inclusion scenarios on the comprehensiveness and results of each overview, examined the patterns and themes that emerged across overviews, identified groups of similar and contrasting overviews, and narratively described these different groups of overviews [13, 26]. We then provided a narrative summary of challenges encountered when making different inclusion decisions in overviews, along with the number of overview topics affected, potential implications, and examples.

## Results

### Description of overview cases

The seven overviews included in this study contained 6–19 SRs (range 0–7 Cochrane SRs, 2–13 non-Cochrane SRs). The SRs had a median publication year of 2008 (range 1989–2013) and a mean AMSTAR score of 7.0/11 (SD 2.8). Compared to the non-Cochrane SRs included in the overviews, the Cochrane SRs were more recent (2010 vs. 2007) and of higher quality (9.6 vs. 5.7). Of the 30 Cochrane SRs, three were new publications and 27 were updates (median 2 updates; range 2–5 updates). Across the overviews, 43% of the included primary studies appeared in multiple SRs (range 23–53% per overview topic), and 53% and 77% were included in Cochrane and non-Cochrane SRs, respectively. Across the overviews, the study overlap between the SRs ranged from slight (CCA 3.3) to high (CCA 14.9). The characteristics of the SRs included in the seven overviews are presented in Table 1. To maintain consistency with subsequent results tables, the table is organized using the categorization scheme described in the next paragraph.

**Table 1:** Characteristics of the systematic reviews included in each overview

Overview topic and SR category	Number of included SRs	Years of publication of SRs, median (range)^a^	AMSTAR score, mean (standard deviation)	Total number of included primary studies	Unique primary studies, *n* and % included in at least one SR^b^	Primary study overlap between SRs (“CCA”)^c^
Overviews for which Cochrane SRs examined all relevant intervention comparisons						
Bronchiolitis	7	2009 (1996–2010)	8.1 (3.0)	55	29	High (14.9)
Cochrane	4	2010 (2009–2010)	10.5 (0.6)	33	26 (90%)	
Non-Cochrane	3	1997 (1996–2004)	5.0 (1.0)	22	13 (45%)	
Croup	6	2008 (1989–2010)	8.3 (3.0)	69	53	Moderate (6.0)
Cochrane	4	2010 (2006–2010)	9.5 (1.9)	51	50 (94%)	
Non-Cochrane	2	1995 (1989–2000)	6.0 (4.2)	18	18 (34%)	
Gastroenteritis	15	2007 (2001–2012)	7.7 (1.8)	228	114	High (13.3)
Cochrane	3	2010 (2006–2010)	10.7 (0.6)	88	88 (77%)	
Non-Cochrane	12	2007 (2001–2012)	6.9 (1.1)	140	82 (72%)	
Overviews for which Cochrane SRs did not examine all relevant intervention comparisons						
Acute asthma	13	2011 (1997–2013)	7.8 (2.0)	82	56	Slight (3.9)
Cochrane	7	2011 (2002–2013)	8.4 (1.8)	48	45 (80%)	
Non-Cochrane	6	2006 (1997–2013)	7.0 (2.0)	34	34 (61%)	
Acute otitis media	15	2009 (1994–2011)	8.1 (2.6)	260	135	Moderate (6.6)
Cochrane	6	2009 (2007–2011)	10.2 (0.8)	87	84 (62%)	
Non-Cochrane	9	2006 (1994–2010)	6.7 (2.5)	173	107 (79%)	
Eczema	19	2007 (2003–2011)	6.6 (2.9)	198	136	Slight (2.5)
Cochrane	6	2007 (2006–2011)	9.3 (1.8)	29	29 (21%)	
Non-Cochrane	13	2008 (2003–2010)	5.4 (2.4)	169	130 (96%)	
Procedural sedation	13	2009 (2004–2013)	3.7 (1.8)	180	85	Moderate (9.3)
Cochrane	0	NA	NA	NA	NA	
Non-Cochrane	13	2009 (2004–2013)	3.7 (1.8)	180	85 (100%)	
All overviews						
Total	88	2008 (1989–2013)	7.0 (2.8)	1072	608	NA
Cochrane	30	2010 (2002–2013)	9.6 (1.6)	336	322 (53%)	
Non-Cochrane	58	2007 (1989–2013)	5.7 (2.3)	736	469 (77%)	

*CCA* corrected covered area, *NA* not applicable, *SR* systematic review

^a^For Cochrane SRs we used the year last assessed as up-to-date

^b^Each primary study was counted only once, regardless of how many SRs included that study

^c^Categorized using published criteria as “slight” (0–5), “moderate” (6–10), “high” (11–15), or “very high” (> 15)

### Effect of different inclusion scenarios on comprehensiveness and results of overviews

When analyzing study results, we identified two distinct groups of overviews that showed similar patterns of outcome data loss and change: overviews for which Cochrane SRs did, and did not, examine all relevant intervention comparisons. All study results are presented according to this grouping. The impact of the different inclusion scenarios on the comprehensiveness and results of overviews is displayed in Table 2, summarized in Table 3, and described below.

**Table 2:** Impact of the full and restricted inclusion scenarios on the comprehensiveness and results of overviews

Overviews and inclusion scenarios^a^	Number of included SRs (% data loss)	Number of intervention comparisons (% data loss)	Number of primary studies included (% data loss)	Number of subjects included (% data loss)	Number of outcomes (L: % outcomes lost; C: % outcomes changed)				
					Primary	Secondary	Adverse effects	Supplemental	Overall
Overviews for which Cochrane SRs examined all relevant intervention comparisons									
Bronchiolitis									
Full scenario	7 (0%)	8 (0%)	29 (0%)	3526 (0%)	13 (0%)	15 (0%)	20 (0%)	–	48 (0%)
Restricted scenario 1	4 (43%)	8 (0%)	26 (10%)	3294 (7%)	L: 1 (8%) C: 0 (0%)	L: 2 (13%) C: 1 (7%)^c^	L: 2 (10%) C: 0 (0%)	–	L + C: 6 (13%)
Restricted scenario 2	Same as restricted scenario 1								
Restricted scenario 3	Same as restricted scenario 1								
Restricted scenario 4	Same as restricted scenario 1								
Croup									
Full scenario	6 (0%)	16 (0%)	53 (0%)	5181 (0%)	31 (0%)	19 (0%)	0 (0%)	–	50 (0%)
Restricted scenario 1	4 (33%)	16 (0%)	50 (6%)	4717 (9%)	L: 0 (0%) C: 0 (0%)	L: 0 (0%) C: 0 (0%)	L: 0 (0%) C: 0 (0%)	–	L + C: 0 (0%)
Restricted scenario 2	Same as restricted scenario 1								
Restricted scenario 3	Same as restricted scenario 1								
Restricted scenario 4	Same as restricted scenario 1								
Gastroenteritis									
Full scenario	15 (0%)	9 (0%)	114 (0%)	14,801 (0%)	6 (0%)	7 (0%)	22 (0%)	–	35 (0%)
Restricted scenario 1	3 (80%)	9 (0%)	88 (23%)	11,147 (25%)	L: 0 (0%) C: 1 (17%)^d^	L: 1 (14%) C: 1 (14%)^c^	L: 5 (23%) C: 3 (14%)^c^	–	L + C: 11 (31%)
Restricted scenario 2	Same as restricted scenario 1								
Restricted scenario 3	3 (80%)	9 (0%)	46 (60%)	6070 (59%)	L: 0 (0%) C: 1 (17%)^d^	L: 0 (0%) C: 2 (29%)^c^	L: 6 (27%) C: 3 (14%)^c^	–	L + C: 12 (34%)
Restricted scenario 4	Same as restricted scenario 1								
Overviews for which Cochrane SRs did not examine all relevant intervention comparisons									
Acute asthma									
Full scenario	13 (0%)	11 (0%)	56 (0%)	5527 (0%)	12 (0%)	29 (0%)	19 (0%)	16 (0%)	76 (0%)
Restricted scenario 1	7 (46%)	9 (18%)	45 (20%)	4521 (18%)	L: 2 (17%) C: 1 (8%)^b^	L: 10 (35%) C: 0 (0%)	L: 5 (26%) C: 1 (5%)^c^	L: 2 (13%) C: 0 (0%)	L + C: 21 (28%)
Restricted scenario 2	9 (30.8%)	11 (0%)	50 (11%)	5023 (9%)	L: 0 (0%) C: 1 (8%)^b^	L: 1 C: 0 (0%)	L: 0 (0%) C: 1 (5%)^c^	L: 0 (0%) C: 0 (0%)	L + C: 3 (4%)
Restricted scenario 3	9 (30.8%)	11 (0%)	49 (13%)	5006 (9%)	L: 0 (0%) C: 1 (8%)^b^	L: 1 C: 0 (0%)	L: 6 (32%) C: 1 (5%)^c^	L: 0 (0%) C: 0 (0%)	L + C: 9 (12%)
Restricted scenario 4	Same as restricted scenario 2								
Acute otitis media									
Full scenario	15 (0%)	18 (0%)	135 (0%)	28,323 (0%)	6 (0%)	22 (0%)	13 (0%)	–	41 (0%)
Restricted scenario 1	6 (60%)	10 (44%)	84 (62%)	21,907 (32%)	L: 0 (0%) C: 0 (0%)	L: 14 (64%) C: 1 (5%)^c^	L: 4 (31%) C: 3 (23%)^cd^	–	L + C: 22 (54%)
Restricted scenario 2	8 (47%)	15 (17%)	117 (13%)	26,261 (7%)	L: 0 (0%) C: 0 (0%)	L: 9 (41%) C: 1 (5%)^c^	L: 3 (23%) C: 3 (23%)^cd^	–	L + C: 16 (39%)
Restricted scenario 3	5 (67%)	18 (0%)	106 (21%)	23,122 (18%)	L: 2 (33%) C: 0 (0%)	L: 6 (27%) C: 3 (14%)^bc^	L: 2 (15%) C: 3 (23%)^cd^	–	L + C: 16 (39%)
Restricted scenario 4	Unable to calculate^e^								
Eczema									
Full scenario	19 (0%)	27 (0%)	136 (0%)	794,014 (0%)	27 (0%)	–	25 (0%)	–	52 (0%)
Restricted scenario 1	6 (68%)	7 (75%)	29 (79%)	11,418 (99%)	L: 20 (74%) C: 2 (7%)^bc^	–	L: 13 (52%) C: 0 (0%)	–	L + C: 35 (67%)
Restricted scenario 2	11 (42%)	22 (19%)	115 (17%)	697,014 (15%)	L: 5 (19%) C: 2 (7%)^bc^	–	L: 13 (52%) C: 0 (0%)	–	L + C: 20 (39%)
Restricted scenario 3	12 (42%)	27 (0%)	133 (15%)	792,721 (4%)	L: 0 (0%) C: 3 (11%)^bcd^	–	L: 22 (88%) C: 0 (0%)	–	L + C: 25 (48%)
Restricted scenario 4	Unable to calculate^e^								
Procedural sedation									
Full scenario	13	–	85	149,088	–	–	–	–	–
Restricted scenario 1	0 (100%)	–	0 (100%)	0 (100%)	–	–	–	–	–
Restricted scenario 2	–	–	–	–	–	–	–	–	–
Restricted scenario 3	–	–	–	–	–	–	–	–	–
Restricted scenario 4	–	–	–	–	–	–	–	–	–

*C* outcome changed, *L* outcome lost, *L+C* outcome lost or changed, *SR* systematic review

^a^ Full scenario: include all Cochrane and non-Cochrane SRs; restricted scenario 1: include only Cochrane SRs; restricted scenarios 2–4: include all non-overlapping SRs, and in the case of overlapping SRs include the Cochrane SR (restricted scenario 2), the most recent SR (restricted scenario 3), or the highest quality SR (restricted scenario 4)

^b^Result assessment changed from “favorable” or “unfavorable” to “neutral”

^c^Result assessment changed from “unknown” to something else

^d^Result assessment changed from “neutral” to “favorable” or “unfavorable”

^e^Two systematic reviews were tied for “highest quality”

**Table 3:** Summary table: amount of outcome data included in each overview when comparing different inclusion scenarios

	Full scenario vs. restricted scenario 1	Restricted scenario 2 vs. 1	Restricted scenario 2 vs. 3	Restricted scenario 2 vs. 4	Restricted scenario 4 vs. 3
Overviews for which Cochrane SRs examined all relevant intervention comparisons					
Bronchiolitis	More	Same	Same	Same	Same
Croup	Same	Same	Same	Same	Same
Gastroenteritis	More	Same	More	Same	More
Overviews for which Cochrane SRs did not examine all relevant intervention comparisons					
Acute asthma	More	More	More	Same	More
Acute otitis media	More	More	Similar	Unknown	Unknown
Eczema	More	More	More	Unknown	Unknown
Procedural sedation	More	Unknown	Unknown	Unknown	Unknown

Full scenario: include all Cochrane and non-Cochrane SRs; restricted scenario 1: include only Cochrane SRs; restricted scenarios 2–4: include all non-overlapping SRs, and in the case of overlapping SRs include the Cochrane SR (restricted scenario 2), the most recent SR (restricted scenario 3), or the highest quality SR (restricted scenario 4). “More” means that more outcome data were included in the first-listed vs. second-listed scenario; “same” means that the same amount of outcome data were included in both scenarios; “similar” means that the same amount of outcome data were included in both scenarios, but the breakdowns differed across primary, secondary, and adverse effects outcomes; “unknown” means that we were unable to calculate the amount of outcome data

#### Overviews for which Cochrane SRs examined all relevant intervention comparisons

In the bronchiolitis, croup, and gastroenteritis overviews, all relevant intervention comparisons were examined in the Cochrane SRs. For the bronchiolitis and gastroenteritis overviews, though all non-Cochrane SRs overlapped with Cochrane SRs, the non-Cochrane SRs sometimes contributed additional primary studies, outcomes, and/or time points that were not included in the Cochrane SRs. Thus, when restricting to Cochrane SRs only (restricted scenario 1), these additional non-Cochrane data, which contributed to 13% (bronchiolitis) and 31% (gastroenteritis) of all outcomes, were lost. When reintroducing all non-overlapping SRs to the Cochrane SRs (restricted scenario 2), these outcome data remained lost. For the croup overview, the non-Cochrane SRs did not contribute any unique outcome data not already contained within the Cochrane SRs, so data loss was 0%. For all three overviews, the outcome data in restricted scenarios 1 and 2 were the same.

For the bronchiolitis and croup overviews, the Cochrane SRs (restricted scenario 2) were always the most recent SRs (restricted scenario 3) and the highest quality SRs (restricted scenario 4), making restricted scenarios 1–4 the same in terms of comprehensiveness and results. For the gastroenteritis overview, the Cochrane SRs were always the highest quality SRs (restricted scenario 4), making restricted scenarios 1, 2, and 4 the same. Including Cochrane SRs (restricted scenarios 1, 2, and 4) compared to the most recent SRs (restricted scenario 3) led to less data loss and change.

#### Overviews for which Cochrane SRs did not examine all relevant intervention comparisons

In the acute asthma, acute otitis media, eczema, and procedural sedation overviews, not all relevant intervention comparisons were examined in the Cochrane SRs. For the acute asthma, eczema, and acute otitis media overviews in particular, when restricting to Cochrane SRs only (restricted scenario 1), the non-Cochrane outcome data, which contributed to 28% (acute asthma), 54% (acute otitis media), and 67% (eczema) of all outcomes, were lost. When reintroducing all non-overlapping SRs to the Cochrane SRs (restricted scenario 2), all non-Cochrane data for unique intervention comparisons were recaptured. In restricted scenario 2, data remained lost or changed for 4% (acute asthma) and 39% (acute otitis media, eczema) of outcomes. Thus, the outcome data in restricted scenario 2 were always more comprehensive than those in restricted scenario 1, and data were recaptured for 86% (acute asthma), 28% (acute otitis media), and 42% (eczema) of lost or changed outcomes. We were unable to extract outcome data for the procedural sedation overview because data for the comparator groups were often unavailable. However, no Cochrane SRs were included in this overview. Thus, in restricted scenario 1, all outcome data would have been lost.

For the acute asthma, acute otitis media, and eczema overviews, including Cochrane SRs (restricted scenario 2) compared to the most recent SRs (restricted scenario 3) led to less or the same amount of data loss and change. For the acute asthma overview, the Cochrane SRs (restricted scenario 2) were always the highest quality SRs (restricted scenario 4), making restricted scenarios 2 and 4 the same in terms of comprehensiveness and results. For the eczema and acute otitis media overviews, we were unable to calculate the amount of data loss and change for restricted scenario 4, because SRs were sometimes “tied” for highest quality. Notably, it was always a Cochrane SR and a most recent SR that were tied for highest quality (Additional file 2). For the procedural sedation overview, it is unclear what would have happened in restricted scenarios 2–4.

### Challenges related to including overlapping SRs in overviews

When conducting the seven overviews and analyzing their outcome data, we identified and documented challenges related to identifying overlapping SRs (two challenges), making different inclusion decisions in overviews (seven challenges), and extracting and analyzing outcome data from overlapping SRs (three challenges). These challenges, along with potential implications and examples, are presented in Table 4 and described below.

**Table 4:** Challenges encountered when including overlapping systematic reviews in overviews

Challenge	Implication	Example
Challenges related to identifying overlapping SRs		
Some non-Cochrane SRs were quite broad and examined all relevant interventions for the condition of interest (3/7 overviews affected).	Overview authors may need to closely examine the results of the SRs to identify all intervention comparisons included within these SRs.	One SR in the acute otitis media overview examined “the comparative effectiveness of [all] different treatment options for treating uncomplicated acute otitis media” and contributed outcome data to ten relevant intervention comparisons.
SRs sometimes referenced only one of multiple related publications. Across SRs, this made multiple related publications hard to identify since they looked like independent publications (2/7 overviews affected).	When looking for overlap among primary studies included in SRs, overview authors should look closely for multiple publications of the same primary study.	In the eczema overview, one Cochrane SR and one non-Cochrane SR included primary study. Examining the “list of excluded references” in the Cochrane SR revealed that these references were duplicate publications of the same primary study.
Challenges related to including all Cochrane and non-Cochrane SRs (full inclusion scenario)		
Some overlapping primary studies included in non-Cochrane SRs were identified by, but excluded from, the Cochrane SRs for being outside the scope or for having methodological deficiencies (6/7 overviews affected).	If researchers agree with the inclusion decisions made in the Cochrane SRs, it may not be appropriate to include this subset of excluded primary studies in the overview.	One non-Cochrane SR in the acute asthma overview included a primary study that was excluded by a similar Cochrane SR due to methodological deficiencies (cross-over design inappropriate for acute asthma).
Challenges related to including only Cochrane SRs (restricted scenario 1)		
Input from a clinical expert was often required to determine whether the Cochrane SRs comprehensively examined all relevant intervention comparisons (6/7 overviews affected).	Clinical experts should be prepared and able to help make methodological decisions related to inclusion of SRs in overviews.	A clinical expert determined that the Cochrane SRs identified for the croup and acute otitis media overviews likely were comprehensive, and were not comprehensive, respectively.
Multiple Cochrane SRs sometimes contributed outcome data to the same comparison (i.e., Cochrane SRs sometimes overlapped) (2/7 overviews affected).	Including Cochrane SRs in overviews may not always eliminate issues related to overlapping SRs. Additional decision rules may be needed to address this situation.	Two Cochrane SRs on epinephrine for treatment of bronchiolitis, and glucocorticoids for treatment of bronchiolitis, each included outcome data for the comparison “epinephrine and glucocorticoid vs. placebo.”
Challenges related to including all non-overlapping SRs, and in the case of overlapping SRs, the Cochrane, most recent, or highest quality SR (restricted scenarios 2–4)		
Not all groups of overlapping SRs included a Cochrane SR (2/7 overviews affected).	Additional decision rules may be needed to capture data from groups of overlapping SRs that do not include a Cochrane SR.	In the eczema overview, two overlapping non-Cochrane SRs (but no Cochrane SRs) provided outcome data on “pet exposure vs. no pet exposure at home.”
Overlapping SRs were sometimes “tied” for most recent year of publication or for highest quality (3/7 overviews affected).	Additional decision rules may be needed to differentiate between SRs with similar publication dates or quality scores.	Because the two most recent overlapping SRs in the acute otitis media overview were both published in 2010, we instead examined the search dates to determine which to include in the overview.
Search dates were not comprehensively or consistently reported in all SRs (6/7 overviews affected).	Using search dates to choose between overlapping SRs published in the same year may not always be possible or straightforward.	When looking at search dates of SRs, the following issues were encountered: exact search dates not reported; month of search not reported; and different search dates reported for different databases.
Conducting quality assessments could be challenging and time-intensive (7/7 overviews affected).	Using methodological quality as an inclusion criterion to choose between groups of overlapping SRs may be more complex than using other decision rules.	For all overview topics, we found that conducting quality assessments was always more time and resource intensive than simply assessing the Cochrane status or year of publication of the SRs.
Challenges related to extracting and analyzing outcome data from overlapping SRs (full inclusion scenario)		
Overlapping SRs sometimes presented the same or similar outcome data in different ways (6/7 overviews affected).	Researchers must decide how best to extract data from overlapping SRs when the same or similar outcome data are reported differently across SRs.	Different SRs sometimes reported different numerators or denominators for the same outcomes, used different types of analysis or statistical methods to analyze the same outcomes, or measured similar outcomes using different definitions, instruments, scales, or time points.
Overlapping SRs sometimes had discordant results for the same outcome (5/7 overviews affected).	Including outcome data from all overlapping SRs may not result in a coherent overall analysis within a single outcome. Reconciling the discordance may require in-depth exploration of the methods and results of the different SRs.	Two SRs in the gastroenteritis overview examined “length of hospital stay” for “oral rehydration therapy vs. intravenous therapy.” One SR contained meta-analyzed data that significantly favored the intervention. The other SR contained narrative data that was not significant.
When including non-Cochrane SRs, data extraction was sometimes difficult due to deficiencies in conduct and reporting of SRs (6/7 overviews affected).	Non-Cochrane SRs with gross deficiencies in conduct and/or reporting may be difficult to include in overviews.	For Cochrane SR, study-level outcome data were always available in well-reported narrative summaries or meta-analyses, but this was not always the case for non-Cochrane SRs.

Identifying groups of overlapping SRs was challenging when the SRs examined all interventions (as opposed to one specific intervention) for the condition of interest and when the primary studies contained within the SRs had multiple related publications that were referenced differently across SRs. Overview authors may need to closely examine the content of the SRs and their included primary studies to accurately assess the extent and nature of the overlap.

All five decisions for SR inclusion presented their own challenges. When including all Cochrane and non-Cochrane SRs in the overviews, many primary studies that were included in the non-Cochrane SRs were identified by, but excluded from, the Cochrane SRs, for being outside their scope or having methodological deficiencies (median 38%; range 8–100%). When including only Cochrane SRs, clinical input was often required to assess whether or not the Cochrane SRs comprehensively examined all relevant intervention comparisons. When including only one SR per group of overlapping SRs, the following challenges were encountered: Cochrane SRs sometimes overlapped; not all groups of overlapping SRs included a Cochrane SR; some groups of overlapping SRs contained multiple SRs that were “tied” for most recent or highest quality; and reporting of SRs sometimes made it challenging to use recency and quality as inclusion criteria. In all cases, overview authors may need additional decision rules to appropriately address these challenges.

Extracting and analyzing outcome data from overlapping SRs often proved challenging. Overlapping SRs often analyzed or presented the same outcome data in different ways or had discordant results for the same outcomes, potentially due to differences in SRs’ inclusion criteria or methods of analysis. Including overlapping SRs in overviews also meant that we encountered older and/or low-quality non-Cochrane SRs that had gross deficiencies in conduct and/or reporting. These commonly encountered challenges may increase the complexity of the data extraction process and make it difficult for overview authors to extract outcome data in a systematic and transparent way.

## Discussion

The current study involved conducting each of seven overviews using five different sets of inclusion criteria, to provide empirical evidence examining the inclusion of overlapping SRs in overviews. This study found that different inclusion decisions led to different amounts of outcome data loss and change across overviews. Specifically, including only Cochrane SRs (i.e., not considering non-Cochrane SRs) often, but not always, led to a loss of outcome data. For groups of overlapping SRs (i.e., when considering both Cochrane and non-Cochrane SRs), selecting the Cochrane SR, as opposed to the most recent or highest quality SR, maximized the amount of outcome data included in the overview. This study also identified challenges associated with identifying, including, and extracting outcome data from overlapping SRs in overviews.

Examining the different inclusion scenarios across overview topics revealed that including only Cochrane SRs, compared to all Cochrane and non-Cochrane SRs, often, but not always, led to a loss of outcome data. The data loss always occurred for one of two reasons. First, when overviews had Cochrane SRs that examined all relevant intervention comparisons, all data loss occurred because the overlapping non-Cochrane SRs contributed additional primary studies, outcomes, and/or time points for existing intervention comparisons. It is unclear whether these additional outcome data are of clinical importance. On the one hand, these results data are lost. In the current study, this led to the complete loss of some outcomes, and changes in the statistical significance of some outcomes. On the other hand, some of these lost outcome data came from primary studies in non-Cochrane SRs that were identified by, but excluded from, the Cochrane SRs, for being outside their scope or having methodological deficiencies. If researchers agree with the inclusion decisions made in the Cochrane SRs, it may not be appropriate to include this subset of excluded primary studies in the overview, especially if these studies are of lower quality or do not increase the certainty of evidence (i.e., GRADE). Second, when overviews had Cochrane SRs that did not examine all relevant intervention comparisons, data loss also occurred because the non-Cochrane SRs contributed outcome data for unique intervention comparisons not examined in any Cochrane SR. These additional data fell within the scope of the overview and were likely of clinical importance, and restricting to only Cochrane SRs led to the exclusion of relevant intervention comparisons from the overview. However, reintroducing all non-overlapping SRs to the Cochrane SRs allowed non-Cochrane data for the non-overlapping intervention comparisons to be recaptured.

Examining the different inclusion scenarios also revealed that for groups of overlapping SRs, selecting the Cochrane SR, as opposed to the most recent or highest quality SR, maximized the amount of outcome data included in the overview. Across overview topics, the Cochrane SRs were sometimes the most recent SRs and were often or always the highest quality SRs. Thus, researchers may often end up selecting Cochrane SRs for inclusion in overviews regardless of which inclusion criteria are used. Further, including Cochrane SRs, even when there are more recent or higher quality non-Cochrane SRs available, may result in more outcome data included in the overview, potentially because Cochrane SRs consistently present raw study-level data in well-reported narrative summaries or meta-analyses. To capture outcome data from groups of overlapping SRs that do not contain a Cochrane SR, researchers may choose to include one of the non-Cochrane SRs. In these cases, our results suggest that selecting the highest quality, as opposed to the most recent, non-Cochrane SR, may minimize data loss.

When conducting the overviews, we often encountered practical challenges related to overlapping SRs. In fact, it was sometimes challenging to simply identify groups of overlapping SRs. We found that overlap can occur at the level of the SRs, but also within SRs at the level of the included primary studies, intervention comparisons, or outcome data. Thus, the issue of “overlap” may be more complex than previously envisioned. There were challenges associated with all five inclusion decisions examined in this study. Notably, many challenges related to extracting and analyzing outcome data from overlapping SRs, especially when multiple SRs contained the same or similar outcome data, and when non-Cochrane SRs were poorly conducted and/or reported. A clear understanding of these challenges, combined with consistent application of appropriate decision rules, can help overview authors successfully manage overlapping SRs in overviews.

The results of this study were used to develop an evidence-based decision tool to help researchers make informed inclusion decisions in overviews. This decision tool is presented and described in a companion paper by Pollock et al. [12]. The tool contains four questions: (1) Do Cochrane SRs likely examine all relevant intervention comparisons?; (2) Do the Cochrane and non-Cochrane SRs overlap?; (3) Do the non-Cochrane SRs overlap with each other; and (4) Are researchers prepared and able to avoid double-counting outcome data from overlapping SRs, by ensuring that each primary study’s outcome data are extracted from overlapping SRs only once? Each yes/no question is answered sequentially, as needed, and the tool is structured so that different answers correspond to appropriate inclusion decisions. Guidance is provided to help researchers answer each question, and the tool provides empirical evidence regarding the impact, advantages, disadvantages, and potential trade-offs of the different inclusion decisions. The decision tool can provide overview authors with the knowledge and means to make informed inclusion decisions in overviews, by helping them determine which inclusion decision may be best suited to their specific situation.

The findings of this study should be considered in light of four methodological considerations. First, this study used one of two standard methods of outcome data analysis that involved extracting and reanalyzing the data from SRs (as opposed to presenting the data exactly as they appear in the SRs) [6]. This was done to avoid double-counting outcome data from multiple overlapping SRs. However, there is currently no evidence regarding whether or to what extent the two methods of outcome data analysis may affect the results of overviews. Second, this study operated under the simplified assumption that within each overview, all outcome data, intervention comparisons, primary studies, and SRs were equally relevant. As judgments about “relevance” would have been difficult to incorporate into the analysis in an objective and systematic way, we weighted all outcome data equally and did not comment on the clinical relevance of the specific data that were lost or changed. For similar reasons, we were also unable to account for potential differences in reporting of SRs (specifically selective outcome reporting) that may have affected the comprehensiveness and results of the overview cases. Third, though we extracted, analyzed, and presented data for a number of potentially relevant variables of interest, we focused our results on the variable that helped explain the different patterns of outcome data loss (i.e., the number of intervention comparisons included in Cochrane and non-Cochrane SRs) [13]. We opted not to discuss the other variables in detail, as they did not contribute to the overall pattern of findings in a cohesive or consistent way. For example, we hypothesized that differences in amounts of primary study overlap may lead to systematic differences in comprehensiveness and results of overviews across various inclusion scenarios, but found that this was not the case. Lastly, the inclusion decisions examined in this study are commonly cited as practical ways to manage overlapping SRs in overviews while avoiding issues related to double-counting outcome data [6, 7, 11]. However, real-life inclusion decisions are not always as straightforward as those examined in this study. For example, there are no accepted criteria for selecting a single SR when two or more SRs are “tied” for highest quality, and researchers may also manage groups of overlapping SRs by choosing to include the “most comprehensive SRs” or the “most relevant SRs.” These subjective decisions were not examined in the current methods study, as they may be operationalized in different ways depending on the author group or overview topic.

As is standard with a multiple case study, we aimed to establish generalizability of our findings by demonstrating replication logic across cases [13]. We described individual cases, looked for patterns across cases, identified similar and contrasting cases, and described groups of similar cases together. Our main study findings remained stable across overviews with a range of characteristics. For example, the overviews included different numbers of SRs (6–19) with various publication dates (1989–2013) and quality scores (1–11 out of 11), had “slight” to “high” primary study overlap between SRs, and had non-Cochrane SRs that contributed 0–100% of outcome data. Achieving replication across multiple cases with different characteristics helps establish robustness of the findings and suggests that the patterns observed within and across cases are coherent, systematically related, and unified [13]. This strengthens the generalizability of the patterns of knowledge gained from the study [13]. However, for coherence, we necessarily used a convenience sample of overviews that posed unique clinical questions within the bounds of certain pre-defined limits. Future research may examine whether or to what extent the observed patterns of findings generalize to a broader group of overviews of healthcare interventions. Findings should not be generalized to overviews that address different clinical questions (e.g., qualitative, diagnostic test accuracy, or prognostic overviews).

Though the number of published SRs will likely keep increasing over time, ongoing developments in the research community may alter the extent and nature of future SR duplication. Core outcome sets for effectiveness trials and for specific health conditions may help offset challenges related to variations in outcome measures across multiple overlapping SRs (www.comet-initiative.org). Perhaps more importantly, prospective systematic review registers—and the increasing expectation for authors to register their protocols—may help reduce the number of overlapping SRs being conducted by different author groups at the same time (www.crd.york.ac.uk/prospero/). These ongoing developments may have the potential to impact methodological decisions relating to inclusion of overlapping SRs in overviews and should be considered going forward.

## Conclusions

There is currently limited guidance available for researchers conducting overviews of healthcare interventions. For example, there are challenges and uncertainty regarding the methods that should be used to manage overlapping SRs in overviews. The current study helps address this gap in guidance by contributing empirical evidence examining the impact of different inclusion decisions on the comprehensiveness and results of overviews. Our results highlight practical challenges related to inclusion of overlapping SRs in overviews and show that different inclusion decisions affect the comprehensiveness and results of overviews in different ways. The results were used to develop an evidence-based decision tool to help researchers make transparent and well-informed inclusion decisions in overviews. This decision too, presented and described in Pollock et al. [12], provides practical guidance for overview authors and warrants further evaluation.

## Additional files

Additional file 1: Inclusion criteria used in each overview, stratified by overview topic. (DOCX 17 kb)

Additional file 2: Comparisons and systematic reviews included in different inclusion scenarios, for each overview topic. (DOCX 54 kb)

## Abbreviations

AMSTAR: A MeaSurement Tool to Assess systematic Reviews
CCA: Corrected covered area
Overview: Overview of reviews
SR: Systematic review
