# Supplementary material for: The impact of different inclusion decisions on the comprehensiveness and complexity of overviews of reviews of healthcare interventions
Source: Syst Rev. 2019 Jan 11;8:18. doi: 10.1186/s13643-018-0914-3 (PMC6329144; doi:10.1186/s13643-018-0914-3)
Supplement: Supplementary file 1 — Inclusion criteria used in each overview, stratified by overview topic. (DOCX 17 kb) [file 13643_2018_914_MOESM1_ESM.docx]

**Additional file 1.** Inclusion criteria used in each overview, stratified by overview topic.

| **Population** | **Intervention** | **Comparator** | **Outcome measures^a^** | **Study design** |
| --- | --- | --- | --- | --- |
| **Acute asthma** | | | | |
| Children aged 0-18 years treated in the ED or equivalent for acute exacerbation of asthma or recurrent wheeze. | Any inhaled short-acting bronchodilator. All doses and frequencies of administration were included. | All comparators. | Primary outcomes: Hospital admission, ED LOS, ICU admission.  Secondary outcomes: Clinical severity scores, vital signs (respiratory rate, heart rate, oxygen saturation).  Adverse effects: Nausea, vomiting, tremor, other general or specific outcomes deemed undesirable.  Supplemental outcomes: PEF, FEV_1_. | SRs of RCTs |
| **Acute otitis media** | | | | |
| Children aged 0-18 years with acute otitis media. | All pharmacological interventions.^b^ | All pharmacological comparators.^b^ | Primary outcome: Pain early in the course of therapy.  Secondary outcomes: Treatment failure (persistence of acute otitis media signs and symptoms) at the end of therapy, recurrence.  Adverse effects: Any. | SRs of RCTs |
| **Bronchiolitis** | | | | |
| Outpatient children with bronchiolitis.^c^ | All interventions. | All comparators. | Primary outcomes: Hospitalization rate on day one, within seven days, at any other time points.  Secondary outcomes: ED LOS, clinical severity score at 60 and 120 minutes.  Adverse effects: Any. | SRs of RCTs |
| **Croup** | | | | |
| Children with croup. | Glucocorticoids, inhaled epinephrine, humidified air or heliox. | Glucocorticoids, inhaled epinephrine, humidified air or heliox. | Primary outcome: Severity of respiratory distress (clinical croup score, clinical improvement).  Secondary outcomes: Hospital admissions, length of stay, re-admissions, risk of intubation. | SRs of RCTs |
| **Eczema** | | | | |
| Children aged 0-18 years at high-risk, and not selected for risk, of developing eczema. | All interventions.^d^ | All comparators.^d^ | Primary outcome: Incidence of eczema or atopic eczema.^e^  Adverse effects: Any | SRs of RCTs and observational studies |
| **Gastroenteritis** | | | | |
| Children aged 0-18 years with acute gastroenteritis. | ORT, anti-emetics and probiotics.^f^ | All comparators.^f^ | Primary outcome: Rate of hospital admission.  Secondary outcomes: Hospital LOS, rate of return visits, administration of IV therapy (due to failure of ORT).  Adverse effects: Any, including dysnatremia (for comparisons involving IV therapy) | SRs of RCTs |
| **Procedural sedation** | | | | |
| Children aged 1 month to 21 years requiring procedure-related sedation in the ED or similar setting. | All doses and routes of administration for: propofol (with or without opioid), ketamine, ketamine/propofol combined, nitrous oxide, and midazolam. | All comparators. | Primary outcome: Adverse effects (any side effect, adverse effect, or adverse event).  Secondary outcomes: Serious interventions for an adverse effect, efficacy (successful completion of the procedure, level/depth of sedation), length of sedation, ED LOS. | SRs of RCTs and observational studies |

ED: emergency department; FEV_1_: forced expiratory volume in one second; ICU: intensive care unit; IgE: immunoglobulin E; IV: intravenous; LOS: length of stay; ORT: oral rehydration therapy; PEF: peak expiratory flow; RCT: randomized controlled trial; SR: systematic review.

^a^ For overviews that did not specify primary outcomes (acute otitis media, bronchiolitis, croup, gastroenteritis), we considered the efficacy outcome listed first in the overview to be the primary outcome and all other efficacy outcomes to be secondary outcomes.

^b^ We excluded comparisons involving bromidoprim (which is not commonly used), and comparisons examined in only one primary study.

^c^ The original overview included outpatients, inpatients, and ICU patients.

^d^ Except for comparisons examined in only one primary study.

^e^ The original overview also included “atopy/IgE sensitization”, “eczema severity”, “time to development of eczema”, “quality of life”, and “healthcare utilization” as secondary outcomes.

^f^ We excluded comparisons involving pyrilamine-pentobarbital, promethazine, and trimethobenzamide (which are not commonly used).
