# Supplementary material for: The impact of different inclusion decisions on the comprehensiveness and complexity of overviews of reviews of healthcare interventions
Source: Syst Rev. 2019 Jan 11;8:18. doi: 10.1186/s13643-018-0914-3 (PMC6329144; doi:10.1186/s13643-018-0914-3)
Supplement: Supplementary file 2 — Comparisons and systematic reviews included in different inclusion scenarios, for each overview topic. (DOCX 54 kb) [file 13643_2018_914_MOESM2_ESM.docx]

**Additional file 2.** Comparisons and systematic reviews included in different inclusion scenarios, for each overview topic.

**Table 1.** Acute asthma.

| **Comparison** | **Systematic reviews** | **Full scenario: Include all Cochrane and non-Cochrane SRs** | **Restricted scenario 1: Include only Cochrane SRs** | **Include all non-overlapping SRs, and for each group of overlapping SRs include the…** | | |
| --- | --- | --- | --- | --- | --- | --- |
|  |  |  |  | **Restricted scenario 2: Cochrane SR** | **Restricted scenario 3: Most recent SR** | **Restricted scenario 4: Highest quality SR** |
| Younger children^a^: SABA *vs.* placebo | Chavasse Ⓒ | 🗸 | 🗸 | 🗸 | 🗸 | 🗸 |
| Younger children: SABA and SAAC *vs.* SABA alone | Everard Ⓒ | 🗸 | 🗸 | 🗸 | 🗸 | 🗸 |
| Younger children: SABA delivered by MDI with spacer or VHC *vs.* nebuliser | Cates ⒸⓇⓆ | 🗸 | 🗸 | 🗸 | 🗸 | 🗸 |
|  | Castro-Rodriguez | 🗸 |  |  |  |  |
| Older children^b^: SABA *vs.* SAAC | Teoh Ⓒ | 🗸 | 🗸 | 🗸 | 🗸 | 🗸 |
| Older children: SABA *vs.* adrenaline | Rodrigo 2006 | 🗸 |  | 🗸 | 🗸 | 🗸 |
| Older children: SABA *vs.* SABA | Jat | 🗸 |  | 🗸 | 🗸 | 🗸 |
| Older children: SABA and SAAC *vs.* SABA alone | Griffiths ⒸⓇⓆ | 🗸 | 🗸 | 🗸 | 🗸 | 🗸 |
|  | Rodrigo 2005 | 🗸 |  |  |  |  |
| Older children: SABA and SAAC *vs.* SAAC alone | Teoh Ⓒ | 🗸 | 🗸 | 🗸 | 🗸 | 🗸 |
| Older children: SABA and MgSO4 *vs.* SABA alone | Powell ⒸⓆ | 🗸 | 🗸 | 🗸 |  | 🗸 |
|  | Shan Ⓡ | 🗸 |  |  | 🗸 |  |
| Older children: SABA delivered by MDI with spacer or VHC *vs.* nebuliser | Cates ⒸⓇⓆ | 🗸 | 🗸 | 🗸 | 🗸 | 🗸 |
|  | Amirav | 🗸 |  |  |  |  |
| Older children: SABA delivered by continuous *vs.* intermittent nebulisation | Camargo Ⓒ | 🗸 | 🗸 | 🗸 | 🗸 | 🗸 |

Ⓒ: Cochrane SR; Ⓡ: Most recent SR; Ⓠ: Highest quality SR; MDI: metered dose inhaler; MgSO_4_: magnesium sulfate; SAAC: short-acting anticholinergic; SABA: short-acting beta agonist; SR: systematic review; VHC: valved holding chamber.

^a^ 0-3 years of age; ^b^ 3-18 years of age.

**Table 2.** Acute otitis media.

| **Comparison** | **Systematic reviews** | **Full scenario: Include all Cochrane and non-Cochrane SRs** | **Restricted scenario 1: Include only Cochrane SRs** | **Include all non-overlapping SRs, and for each group of overlapping SRs include the…** | | |
| --- | --- | --- | --- | --- | --- | --- |
|  |  |  |  | **Restricted scenario 2: Cochrane SR** | **Restricted scenario 3: Most recent SR** | **Restricted scenario 4: Highest quality SR** |
| Any antibiotic *vs.* placebo | Sanders Ⓒ | 🗸 | 🗸 | 🗸 |  |  |
|  | Damoiseaux | 🗸 |  |  |  |  |
|  | Del Mar | 🗸 |  |  |  |  |
|  | Rosenfeld | 🗸 |  |  |  |  |
|  | Rovers | 🗸 |  |  |  |  |
|  | Shekelle ⓇⓆ | 🗸 |  |  | 🗸 | 🗸 |
|  | Vouloumanou | 🗸 |  |  |  |  |
| Short-course antibiotic (single dose azythromycin) *vs.* long-course antibiotic | Korzyskyj ⒸⓆ | 🗸 | 🗸 | 🗸 |  | 🗸 |
|  | Courter | 🗸 |  |  |  |  |
|  | Gulani | 🗸 |  |  |  |  |
|  | Shekelle ⓇⓆ | 🗸 |  |  | 🗸 | 🗸 |
| Short-course antibiotic (3-5 days azythromycin) *vs.* long-course antibiotic | Korzyskyj ⒸⓆ | 🗸 | 🗸 | 🗸 |  | 🗸 |
|  | Courter | 🗸 |  |  |  |  |
|  | Gulani | 🗸 |  |  |  |  |
|  | Ioannidis | 🗸 |  |  |  |  |
|  | Shekelle ⓇⓆ | 🗸 |  |  | 🗸 | 🗸 |
| Short-course antibiotic (intramuscular ceftriaxone) *vs.* long-course antibiotic | Korzyskyj ⒸⓆ | 🗸 | 🗸 | 🗸 |  | 🗸 |
|  | Gulani | 🗸 |  |  |  |  |
|  | Shekelle ⓇⓆ | 🗸 |  |  | 🗸 | 🗸 |
| Any other short-course antibiotic (> 48 hours) *vs.* long-course antibiotic | Korzyskyj ⒸⓆ | 🗸 | 🗸 | 🗸 |  | 🗸 |
|  | Gulani | 🗸 |  |  |  |  |
|  | Shekelle ⓇⓆ | 🗸 |  |  | 🗸 | 🗸 |
| Amoxicillin with(out) clavulanate administered once or twice *vs.* three times daily | Thanaviratananich Ⓒ | 🗸 | 🗸 | 🗸 |  |  |
|  | Shekelle ⓇⓆ | 🗸 |  |  | 🗸 | 🗸 |
| Amoxicillin with(out) clavulanate *vs.* macrolide | Courter | 🗸 |  |  |  |  |
|  | Shekelle ⓇⓆ | 🗸 |  |  | 🗸 | 🗸 |
| Amoxicillin with(out) clavulanate *vs.* cephalosporin | Rosenfeld | 🗸 |  |  |  |  |
|  | Shekelle ⓇⓆ | 🗸 |  |  | 🗸 | 🗸 |
| Aminopenicillin *vs.* penicillin with(out) sulfisoxazole | Rosenfeld | 🗸 |  | 🗸 | 🗸 | 🗸 |
| Aminopenicillin *vs.* trimethoprim-sulfamethoxazole | Rosenfeld | 🗸 |  | 🗸 | 🗸 | 🗸 |
| Aminopenicillin *vs.* erythromycin | Rosenfeld | 🗸 |  | 🗸 | 🗸 | 🗸 |
| Erythromycin with sulfisoxazole *vs.* cephalosporin | Rosenfeld | 🗸 |  | 🗸 | 🗸 | 🗸 |
| Cefaclor vs. other cephalosporin | Rosenfeld | 🗸 |  |  |  |  |
|  | Shekelle ⓇⓆ | 🗸 |  |  | 🗸 | 🗸 |
| Cefdinir administered once *vs.* twice daily | Shekelle | 🗸 |  | 🗸 | 🗸 | 🗸 |
| Delayed *vs.* immediate antibiotic | Sanders Ⓒ | 🗸 | 🗸 | 🗸 |  |  |
|  | Spurling Ⓒ | 🗸 | 🗸 | 🗸 |  |  |
|  | Rovers | 🗸 |  |  |  |  |
|  | Shekelle ⓇⓆ | 🗸 |  |  | 🗸 | 🗸 |
|  | Vouloumanou | 🗸 |  |  |  |  |
| Delayed *vs.* no antibiotic | Spurling Ⓒ | 🗸 | 🗸 | 🗸 | 🗸 | 🗸 |
| Decongestant and/or antihistamine *vs.* none | Coleman Ⓒ | 🗸 | 🗸 | 🗸 | 🗸 | 🗸 |
| Topical analgesia *vs.* placebo | Foxlee Ⓒ | 🗸 | 🗸 | 🗸 | 🗸 | 🗸 |

Ⓒ: Cochrane SR; Ⓡ: Most recent SR; Ⓠ: Highest quality SR; SR: systematic review.

**Table 3.** Bronchiolitis.

| **Comparison** | **Systematic reviews** | **Full scenario: Include all Cochrane and non-Cochrane SRs** | **Restricted scenario 1: Include only Cochrane SRs** | **Include all non-overlapping SRs, and for each group of overlapping SRs include the…** | | |
| --- | --- | --- | --- | --- | --- | --- |
|  |  |  |  | **Restricted scenario 2: Cochrane SR** | **Restricted scenario 3: Most recent SR** | **Restricted scenario 4: Highest quality SR** |
| Glucocorticoid *vs.* placebo | Fernandes ⒸⓇⓆ | 🗸 | 🗸 | 🗸 | 🗸 | 🗸 |
|  | King | 🗸 |  |  |  |  |
| Epinephrine *vs.* placebo | Hartling Ⓒ | 🗸 | 🗸 | 🗸 | 🗸 | 🗸 |
| Epinephrine and glucocorticoid *vs.* placebo | Fernandes Ⓒ | 🗸 | 🗸 | 🗸 |  |  |
|  | Hartling ⒸⓇⓆ | 🗸 | 🗸 | 🗸 | 🗸 | 🗸 |
| Epinephrine *vs.* bronchodilator | Hartling ⒸⓇⓆ | 🗸 | 🗸 | 🗸 | 🗸 | 🗸 |
|  | King | 🗸 |  |  |  |  |
| Glucocorticoid *vs.* epinephrine | Fernandes Ⓒ | 🗸 | 🗸 | 🗸 |  |  |
|  | Hartling ⒸⓇⓆ | 🗸 | 🗸 | 🗸 | 🗸 | 🗸 |
| Glucocorticoid and bronchodilator *vs.* placebo | Fernandes Ⓒ | 🗸 | 🗸 | 🗸 | 🗸 | 🗸 |
| Bronchodilator *vs.* placebo | Gadomski ⒸⓇⓆ | 🗸 | 🗸 | 🗸 | 🗸 | 🗸 |
|  | Flores | 🗸 |  |  |  |  |
|  | Kellner | 🗸 |  |  |  |  |
|  | King | 🗸 |  |  |  |  |
| 3% hypertonic saline *vs.* 0.9% saline | Zhang 2008 Ⓒ | 🗸 | 🗸 | 🗸 | 🗸 | 🗸 |

Ⓒ: Cochrane SR; Ⓡ: Most recent SR; Ⓠ: Highest quality SR; SR: systematic review.

**Table 4.** Croup.

| **Comparison** | **Systematic reviews** | **Full scenario: Include all Cochrane and non-Cochrane SRs** | **Restricted scenario 1: Include only Cochrane SRs** | **Include all non-overlapping SRs, and for each group of overlapping SRs include the…** | | |
| --- | --- | --- | --- | --- | --- | --- |
|  |  |  |  | **Restricted scenario 2: Cochrane SR** | **Restricted scenario 3: Most recent SR** | **Restricted scenario 4: Highest quality SR** |
| Glucocorticoid *vs.* placebo | Russell ⒸⓇⓆ | 🗸 | 🗸 | 🗸 | 🗸 | 🗸 |
|  | Griffin | 🗸 |  |  |  |  |
|  | Kairys | 🗸 |  |  |  |  |
| Dexamethasone *vs.* budenoside | Russell ⒸⓇⓆ | 🗸 | 🗸 | 🗸 | 🗸 | 🗸 |
|  | Griffin | 🗸 |  |  |  |  |
| Dexamethasone and budenoside *vs.* dexamethasone | Russell ⒸⓇⓆ | 🗸 | 🗸 | 🗸 | 🗸 | 🗸 |
|  | Griffin | 🗸 |  |  |  |  |
| Dexamethasone and budenoside *vs.* budenoside | Russell Ⓒ | 🗸 | 🗸 | 🗸 | 🗸 | 🗸 |
| Dexamethasone *vs.* betamethasone | Russell Ⓒ | 🗸 | 🗸 | 🗸 | 🗸 | 🗸 |
| Dexamethasone vs. prednisolone | Russell Ⓒ | 🗸 | 🗸 | 🗸 | 🗸 | 🗸 |
| Oral dexamethasone *vs.* intramuscular dexamethasone | Russell Ⓒ | 🗸 | 🗸 | 🗸 | 🗸 | 🗸 |
| Dexamethasone (0.30 mg/kg) *vs.* dexamethasone (0.15 mg/kg) | Russell Ⓒ | 🗸 | 🗸 | 🗸 | 🗸 | 🗸 |
| Dexamethasone (0.60 mg/kg) *vs.* dexamethasone (0.30 mg/kg) | Russell Ⓒ | 🗸 | 🗸 | 🗸 | 🗸 | 🗸 |
| Dexamethasone (0.60 mg/kg) *vs.* dexamethasone (0.15 mg/kg) | Russell Ⓒ | 🗸 | 🗸 | 🗸 | 🗸 | 🗸 |
| Glucocorticoid *vs.* epinephrine | Russell Ⓒ | 🗸 | 🗸 | 🗸 | 🗸 | 🗸 |
| Epinephrine *vs.* placebo | Bjornson Ⓒ | 🗸 | 🗸 | 🗸 | 🗸 | 🗸 |
| Racemic epinephrine *vs.* L-epinephrine | Bjornson Ⓒ | 🗸 | 🗸 | 🗸 | 🗸 | 🗸 |
| Epinephrine with IPPB *vs.* epinephrine without IPPB | Bjornson Ⓒ | 🗸 | 🗸 | 🗸 | 🗸 | 🗸 |
| Humidified air *vs.* no treatment | Moore Ⓒ | 🗸 | 🗸 | 🗸 | 🗸 | 🗸 |
| Heliox *vs.* 30% oxygen | Vorweck Ⓒ | 🗸 | 🗸 | 🗸 | 🗸 | 🗸 |

Ⓒ: Cochrane SR; Ⓡ: Most recent SR; Ⓠ: Highest quality SR; IPPB: intermittent positive pressure breathing; SR: systematic review.

**Table 5.** Eczema.

| **Comparison** | **Systematic reviews** | **Full scenario: Include all Cochrane and non-Cochrane SRs** | **Restricted scenario 1: Include only Cochrane SRs** | **Include all non-overlapping SRs, and for each group of overlapping SRs include the…** | | |
| --- | --- | --- | --- | --- | --- | --- |
|  |  |  |  | **Restricted scenario 2: Cochrane SR** | **Restricted scenario 3: Most recent SR** | **Restricted scenario 4: Highest quality SR** |
| EBF for at least 6 months *vs.* introduction of solids at 3-6 months | Kramer 2002 Ⓒ | 🗸 | 🗸 | 🗸 |  |  |
|  | Schneider Chafen ⓇⓆ | 🗸 |  |  | 🗸 | 🗸 |
|  | Tarini | 🗸 |  |  |  |  |
| EBF for at least 3 months *vs.* partial breastfeeding | Yang | 🗸 |  | 🗸 | 🗸 | 🗸 |
| EBF for at least 3 months *vs.* cow’s milk formula | Hanifin | 🗸 |  |  |  |  |
|  | Hoare Ⓠ | 🗸 |  |  |  | 🗸 |
|  | Yang Ⓡ | 🗸 |  |  | 🗸 |  |
| EBF for at least 3 months *vs.* soy formula | Hanifin Ⓡ | 🗸 |  |  | 🗸 |  |
|  | Hoare Ⓠ | 🗸 |  |  |  | 🗸 |
| Hydrolysed formula *vs.* cow’s milk formula | Osborn 2006a ⒸⓆ | 🗸 | 🗸 | 🗸 |  | 🗸 |
|  | Alexander | 🗸 |  |  |  |  |
|  | Hanifin | 🗸 |  |  |  |  |
|  | Hoare | 🗸 |  |  |  |  |
|  | Schneider Chafen | 🗸 |  |  |  |  |
|  | Szajewska ⓇⓆ | 🗸 |  |  | 🗸 | 🗸 |
| Extensively hydrolysed formula *vs.* partially hydrolysed formula | Osborn 2006a ⒸⓆ | 🗸 | 🗸 | 🗸 |  | 🗸 |
|  | Hill | 🗸 |  |  |  |  |
|  | Hoare | 🗸 |  |  |  |  |
|  | Szajewska ⓇⓆ | 🗸 |  |  | 🗸 | 🗸 |
| Soy formula *vs.* cow’s milk formula | Osborn 2006b ⒸⓇⓆ | 🗸 | 🗸 | 🗸 | 🗸 | 🗸 |
|  | Hanifin | 🗸 |  |  |  |  |
|  | Hoare | 🗸 |  |  |  |  |
| Soy formula *vs.* hydrolysed formula | Hill Ⓡ | 🗸 |  |  | 🗸 |  |
|  | Hoare Ⓠ | 🗸 |  |  |  | 🗸 |
| Amino acid-based formula *vs.* hydrolysed formula | Hill | 🗸 |  | 🗸 | 🗸 | 🗸 |
| Amino acid-based formula *vs.* soy formula | Hill | 🗸 |  | 🗸 | 🗸 | 🗸 |
| Maternal antigen avoidance *vs.* standard diet | Kramer 2006 ⒸⓇⓆ | 🗸 | 🗸 | 🗸 | 🗸 | 🗸 |
|  | Hoare | 🗸 |  |  |  |  |
|  | Schneider Chafen | 🗸 |  |  |  |  |
| Omega 3 fatty-acid supplementation *vs.* placebo | Anandan Ⓠ | 🗸 |  |  |  | 🗸 |
|  | Kremmyda Ⓡ | 🗸 |  |  | 🗸 |  |
| Omega 6 fatty-acid supplementation *vs.* placebo | Anandan | 🗸 |  | 🗸 | 🗸 | 🗸 |
| High maternal fish intake *vs.* low or no maternal fish intake | Kremmyda | 🗸 |  | 🗸 | 🗸 | 🗸 |
| High infant fish intake *vs.* low or no infant fish intake | Kremmyda | 🗸 |  | 🗸 | 🗸 | 🗸 |
| Prebiotic *vs.* no prebiotic | Osborn 2007a Ⓒ | 🗸 | 🗸 | 🗸 | 🗸 | 🗸 |
| Probiotic *vs.* no probiotic | Osborn 2007b ⒸⓆ | 🗸 | 🗸 | 🗸 |  | 🗸 |
|  | Flohr | 🗸 |  |  |  |  |
|  | Hanifin | 🗸 |  |  |  |  |
|  | Lee | 🗸 |  |  |  |  |
|  | Schneider Chafen Ⓡ | 🗸 |  |  | 🗸 |  |
| Daycare *vs.* no daycare | Flohr | 🗸 |  | 🗸 | 🗸 | 🗸 |
| Living on a farm *vs.* not living on a farm | Flohr | 🗸 |  | 🗸 | 🗸 | 🗸 |
| Pet exposure at home *vs.* no pet exposure at home | Flohr Ⓠ | 🗸 |  |  |  | 🗸 |
|  | Langan Ⓡ | 🗸 |  |  | 🗸 |  |
| Endotoxin exposure *vs.* no endotoxin exposure | Flohr | 🗸 |  | 🗸 | 🗸 | 🗸 |
| Childhood infection *vs.* no childhood infection | Flohr | 🗸 |  | 🗸 | 🗸 | 🗸 |
| Endoparasites *vs.* no endoparasites | Flohr | 🗸 |  | 🗸 | 🗸 | 🗸 |
| Tuberculin response *vs.* no tuberculin response | Flohr | 🗸 |  | 🗸 | 🗸 | 🗸 |
| BCG vaccination *vs.* no BCG vaccination | Flohr | 🗸 |  | 🗸 | 🗸 | 🗸 |
| Childhood vaccination *vs.* no childhood vaccination | Flohr | 🗸 |  | 🗸 | 🗸 | 🗸 |
| Childhood antibiotics *vs.* no childhood antibiotics | Flohr | 🗸 |  | 🗸 | 🗸 | 🗸 |

Ⓒ: Cochrane SR; Ⓡ: Most recent SR; Ⓠ: Highest quality BCG: Bacille Calmette-Guerin; EBF: exclusive breastfeeding; SR: systematic review.

^a^ Osborn 2006a was included instead of Szajewska because it was more comprehensive (Osborn 2006a examined all hydrolysed formulas, whereas Szajewska examined only partially hydrolysed formulas).

**Table 6.** Gastroenteritis.

| **Comparison** | **Systematic reviews** | **Full scenario: Include all Cochrane and non-Cochrane SRs** | **Restricted scenario 1: Include only Cochrane SRs** | **Include all non-overlapping SRs, and for each group of overlapping SRs include the…** | | |
| --- | --- | --- | --- | --- | --- | --- |
|  |  |  |  | **Restricted scenario 2: Cochrane SR** | **Restricted scenario 3: Most recent SR** | **Restricted scenario 4: Highest quality SR** |
| ORT *vs.* IV therapy | Hartling ⒸⓇⓆ | 🗸 | 🗸 | 🗸 | 🗸 | 🗸 |
|  | Fonseca | 🗸 |  |  |  |  |
| Oral ondansetron *vs.* placebo | Fedorowicz ⒸⓇⓆ | 🗸 | 🗸 | 🗸 | 🗸 | 🗸 |
|  | DeCamp | 🗸 |  |  |  |  |
|  | Szajewska 2007a | 🗸 |  |  |  |  |
| IV ondansetron *vs.* placebo | Fedorowicz ⒸⓇⓆ | 🗸 | 🗸 | 🗸 | 🗸 | 🗸 |
|  | DeCamp | 🗸 |  |  |  |  |
|  | Szajewska 2007a | 🗸 |  |  |  |  |
| IV ondansetron *vs.* dexamethasone | Fedorowicz Ⓒ | 🗸 | 🗸 | 🗸 | 🗸 | 🗸 |
| IV ondansetron *vs.* metoclopramide | Fedorowicz Ⓒ | 🗸 | 🗸 | 🗸 | 🗸 | 🗸 |
| Dimenhydrinate *vs.* placebo | Fedorowicz Ⓒ | 🗸 | 🗸 | 🗸 | 🗸 | 🗸 |
| Dexamethasone *vs.* placebo | Fedorowicz ⒸⓇⓆ | 🗸 | 🗸 | 🗸 | 🗸 | 🗸 |
|  | DeCamp | 🗸 |  |  |  |  |
| Metoclopramine *vs.* placebo | Fedorowicz ⒸⓇⓆ | 🗸 | 🗸 | 🗸 | 🗸 | 🗸 |
|  | DeCamp | 🗸 |  |  |  |  |
| Probiotics *vs.* placebo | Allen ⒸⓆ | 🗸 | 🗸 | 🗸 |  | 🗸 |
|  | Chmielewska | 🗸 |  |  |  |  |
|  | Dinleyici Ⓡ | 🗸 |  |  |  |  |
|  | Huang | 🗸 |  |  |  |  |
|  | McFarland | 🗸 |  |  |  |  |
|  | Salari Ⓡ | 🗸 |  |  | 🗸^a^ |  |
|  | Szajewska 2001 | 🗸 |  |  |  |  |
|  | Szajewska 2007b | 🗸 |  |  |  |  |
|  | Szajewska 2007c | 🗸 |  |  |  |  |
|  | Van Neil | 🗸 |  |  |  |  |

Ⓒ: Cochrane SR; Ⓡ: Most recent SR; Ⓠ: Highest quality SR; IV: intravenous; ORT: oral rehydration therapy; SR: systematic review.

^a^ Salari was included instead of Dinleyici because it was more comprehensive (Salari examined all probiotics, whereas Dinleyici examined only one specific strain of probiotic).
